# Clinicohematological Profiles of Hospitalized Patients with Dengue in Kolkata in 2012 Epidemic, West Bengal

**Published:** 2014-09

**Authors:** Ashis Kumar Saha, Gautam Chatterjee, Subhas Chandra Hazra

**Affiliations:** General Medicine, Kali Pada Chowdhury Medical College and Hospital, Jadavpur, Kolkata, West Bengal, India

**Keywords:** Dengue, Epidemic, India

## Abstract

Dengue usually presents itself with subclinical or mild infection to full blown dengue fever (DF) to dengue hemorrhagic fever (DHF) and dengue shock syndrome (DSS). In Kolkata, dengue started in 1824 followed by five epidemics that occurred in 1836, 1906, 1911, 1923 and 2005. The aim of this investigation is to study the clinicohematological correlation of all patients with respect to their gender that were admitted to “Kali Pada Chowdhury Medical College and Hospital” during 2012 epidemic. Amongst a total of 1237 dengue patients (either dengue Nonstructural protein1 antigen or dengue Immunoglobulin M positive) that were admitted to the hospital, 11 patients died within 48 hours of admission; hence they have been excluded from the study. DHF patients were divided into males and females. During admission, proper history, physical examinations with necessary hematological investigations were performed and repeated again after 24-48 hours. After collection of all the reports, correlations of the collected data were carried out. 170 and 1056 patients were diagnosed with DF and DHF respectively; significant symptoms and signs were headache, backache/myalgia, nausea/vomiting, loose motion and anorexia hepatomegaly. Hemoglobin level was low in females, leucopenia observed in 79.52% patients and thrombocytopenia seen in 57.58% and 86.13% patients during and 24-48 hours after admission respectively. 96 and 97 DHF patients showed evidences of ascites and plural effusion respectively. In 2012 epidemic, 86.13% patients suffered from DHF, headache, backache, nausea/vomiting, loose motion and anorexia were predominant symptoms. Significant number of patients had leucopenia; only few showed evidence of plasma leakage.

## Introduction

Dengue, an Arboviral infection, has a wide spectrum of clinical presentation, from subclinical infection or full blown dengue fever (DF) to severe form of disease, i.e. DHF and DSS. The word “dengue” is derived from the Swahili phrase Ka-dinga pepo which means “cramp like seizure”. Aedes aegypti and Ades Albopictus mosquitoes are responsible for the spread of dengue fever. Its virus has been subdivided into four subtypes, namely DV-1, DV-2, DV-3 and DV-4. The WHO classified dengue fever as undifferentiated fever, dengue fever and dengue hemorrhagic fever. First clinically recognised dengue epidemic occurred more or less simultaneously across Asia, Africa and North America in 1780. However, Benjamin Rush stated the first clinical case report of 1780’s epidemic in Philadelphia in 1789. Dengue virus was first isolated in Kolkata in 1944 from serum of US soldiers. In Madras (now Chennai), the first clinical dengue fever epidemic occurred in the year 1780, but Kolkata and Eastern Coast of India recorded its first virologically proved dengue fever in 1963-64.^1^ First major epidemic of DHF in 1953-54 was in Philippines and despite the presence of all the risk factors, it widely spread into the adjoining countries surrounding India. Ultimately it started creeping into India in 1988.^2^ Since then, major epidemics of DHF/DSS occurred in and around Delhi and Lucknow in 1996 and then spread all over the country. In Kolkata, dengue was first documented in 1824, since then several epidemics occurred in Kolkata in 1836, 1906, 1911, 1923 and 2005. But in 2012, large numbers of serologically proved dengue patients were admitted to different hospitals in Kolkata.

With regards to the rates of infection and severity of the diseases, male-female distinction is very important for public health control programs. In different countries, surveillance data suggests large variations in male-female ratio in dengue infection. It is well known that in many Asian communities, low female incidence may be due to the statistical artefact. This could be as a result of low reporting incidences, the tendency of traditional practitioners in offering inappropriate care for women as well as low incidence of female infection due to their home-staying and less exposure. However, since 1970’s, males are responsible for milder disease whereas females account for more severe illness. The aim of this study is to present clinicopathological profiles of admitted patients throughout their hospital-stay till recovery during 2012 epidemic.

## Patients and Methods

Initially, permission from Ethical Committee was obtained. A total of 1237 patients were admitted to “Kali Pada Chowdhury Medical College and hospital” from the general community during 1^st^ August to 31^st^ October 2012. Immediately after admission, informed consent was obtained from patients. Blood samples were tested for Non Structural Protein1 (Ns1) antigen and Immunoglobulin M (IgM) dengue antibody (Mac ELISA manufactured by Panbio diagnostics). Eleven serologically proved dengue patients died within the first 24 to 48 hours and rest of the 1226 patients recovered from the hospital. Amongst the eleven deceased patients, 6 were >18-40 years, 3 patients >60 years and the remaining 2 patients between >40-60 years. Four patients died due to acute encephalopathy (CT scan showed dilated CSF spaces with severe brain edema) of which 2 had associated severe intractable dyselectrolytemia related with sepsis. Two patients died due to acute severe hepatocellular failure (having severely raised SGPT, INR) where one associated with acute renal failure (Creatinine 8.6mg %) and the other had severe vaginal bleeding (very raised APTT). Two patients died due to uncontrollable severe hypotension with shock; one individual had severe sequestration of fluid in the third spaces and the other had severe uncontrollable gastrointestinal bleeding (INR>2.5) with prerenal azotemia. Two patients died due to ARDS with type 2 respiratory failure. Only one patient died due to acute cardiomyopathy with cardiac dysrrhythmia (ventricular tachycardia).

After admission, thorough history-taking, general and systemic examinations were performed on the 1226 patients. Their background history revealed headache, backache, nausea/vomiting, loose motion, pain abdomen, cough, arthralgia, bleeding, rash and urinary tract infection as predominant symptoms. Physical examinations demonstrated hepatomegaly, evidence of plasma leakage such as ascites, pleural effusion and petechial rashes. Blood samples were sent for hematological and biochemical examinations during and 48 hours after admission, except for those 11 cases (excluded from our study), where platelet counts were below 100000/cc, daily platelet count were advised till the rising trend towards normal. Ultrasonographies (USG) of abdomen and chest x-ray were done to detect the peritoneal and plural involvement. The data was compared according to gender, different presenting symptoms, total WBC count, platelet count, peritoneal and plural involvement in all age groups between males and females. These patients recovered from the ailments within 1 to 2 weeks by conservative measures. Patients with platelet count less than 20000/cc, received platelet transfusion, which showed evidences of bleeding from different sites administered whole blood along with platelets. Again, patients with platelet count between 20000/cc to 40000/cc with evidences of bleeding received platelet, whereas other non-bleeder patients with platelet count between 20000/cc to 40000/cc were under strict follow-up. Also patients with platelet count more than 40000/cc were under strict follow-up. Otherwise, all patients received the symptomatic measures, such as plenty of intravenous fluids, occasionally antibiotics and antipyretics. The frequencies of two samples (males and females) for different items were compared at selected confidence level of 95%, extracted ‘z’ value and probability value (P value) for confidence interval 1.960.

*Statistical Method Used*

1. For significance of percentages, Z values (normal deviates) have been calculated. P value indicates the maximum probability for a given level of significance.
2. 95% CI for difference of percentage:
3. (p_1_-p_2_)±1.96SE (p_1_-p_2_), where SE (p_1_-p_2_)=√ [{p_1_ (1-p_1_)∕n_1_}+{p_2 _(1-p_2_)∕n_2_}]
4. Chi-square test has been used with three degrees of freedom for table 1 to show significance of association of affected cases according to types of symptoms, sign and hematological investigations.

**Table 1:** Comparison of symptoms, hematological investigations in male and female dengue patients

**Itmes**	**Sex**			**Total patients**	**% of patients**
	**Male (591)**	**P value**	**Female (635)**		
Headache	247 (41.79%)	NS	264 (41.57%)	511	41.68
Backache	254 (42.97%)	NS	274 (43.14%)	528	43.06
Nausea/vomiting	190 (32.14%)	0.00	338 (53.22%)	528	43.06
Loose motion	145 (24.534%)	NS	179 (28.18%)	324	26.42
Pain abdomen	40 (6.768%)	NS	42 (6.616%)	82	6.68
Cough	13 (2.19%)	NS	15 (2.36%)	28	2.28
Arthritis	36 (6.09%)	NS	29 (4.56%)	65	5.30
UTI	6 (0.061%)	NS	12 (1.88%)	18	1.46
Anorexia	216 (36.548%)	NS	206 (32.44%)	422	34.42
Bleeding	42 (7.10%)	NS	44 (6.92%)	86	7.01
Rash	58 (9.18%)	NS	57 (8.97%)	115	9.38
Afebrile	343 (58.03%)	NS	352 (55.43%)	695	56.68
*Hematology*					
Total count					
<1000-3000	232 (39.25%)	NS	222 (34.96%)	454	37.03
>3000-5000	262 (44.33%)	NS	259 (40.78%)	521	42.49
>5000	131 (22.16%)	NS	122 (19.21%)	253	20.63
Hemoglobin					
>7-9	12 (2.03%)	0.00	91 (14.33%)		
>9	578 (97.80%)	0.00	540 (85.03%)		
Hematocrit					
20-30	103 (17.42%)	0.00	316 (49.76%)	419	34.17
>30-35	347 (58.71%)	0.00	281 (44.25%)	628	51.22
>35-40	115 (19.45%)	0.00	31 (4.88%)	146	11.90
>40	26 (4.39%)	0.00	7 (1.10%)	33	2.69

## Results

1190 patients were dengue NS1 antigen positive and the remaining 36 patients were dengue antibody IgM positive. Total DF patients were 170, whereas, DHF patients were 1056. Nausea/vomiting were statistically significant (P=0.00001) in females. Males significantly showed raised Hematocrit of >30 to 40 (P=0.00001), >40 (P=0.0002) and hemoglobin of >9 (P=0.00001), whereas female significantly showed hemoglobin level of >7-9 gm% (P=0.00001). During admission, 706 dengue patients (57.58%) showed thrombocytopenia. But within 2 days of infection, 350 more patients showed thrombocytopenia, thus increasing the number of DHF patients to 1056 (86.13%). Male and female patients significantly showed platelet count of >25000-40000/cc (P=0.0422) and >40000-100000/cc (P=0.0002) during admission respectively. Similarly throughout the admission period, male and females patients significantly showed platelet count of >25000-40000/cc (P=0.0288) and >40000-100000/cc (P=0.0066) respectively. During and throughout the admission, platelet value (<15000-25000) showed no significant difference between males and females. But there was no statistical difference in hepatic and serosal involvement in males and females.

## Discussion

According to the WHO criteria, all the 1237 cases were confirmed DF, amongst them, 170 cases were uncomplicated dengue fever (platelet>100000/cc) and the rest of the 1067 cases (including 11 death cases) were DHF. In a study^3^ done by Karoli et al. most common symptoms were headache (76%), abdominal pain (63%), vomiting (58%) and rash (26%). Laboratory examination showed leucopenia (89%) and thrombocytopenia (92%). In the present study, the most common symptoms were headache (41.68%), backache/myalgia (43.06%), nausea/vomiting (43.06% with preponderance in female) and loose motion (26.42%). Most important point in this study was that 57.20% of patients were admitted in 2^nd^ stage (critical phase), whereas two days after admission, this percentage was increased to 86.13% (platelet<100000/cc).

There exists few available hospital-based data showing male-female distribution in dengue infection. Three independent studies in India and Singapore showed affected males twice more common than females. A hospital based study in Delhi in 1996; showed male to female ratio was 2.5:1. According to Bangladesh study, male to female ratio in 1997 epidemic was 1.5:1, but there was no gender predilection in 2000. Studies in South America showed in a typical dengue fever, male to female ratio was 0.65:1.^4^ This study showed male to female ratio was 1:1.08 with little edging in female, but case fatality ratio was significant in females (i.e. 1:2.7, amongst 11 deceased patients there were 3 males and 8 females). Similar statistics is also shown in two Asian studies by Kabra^5^ and Sarkar.^1^ Case fatality ratio in females is due to their more competent immune responses, which is responsible for greater production of cytokines and increased permeability of capillary bed as suggested by Halstead.^6^

Dengue fever was originally well-known as childhood disease in the South East Asia. However, as shown in several studies in Latin America and South East Asia, there was gradual inclination towards higher age groups in DHF since early 1980’s. The earliest studies were by Guzman^7^ (1981) in Cuba and Rigau-Perez^8^ in Puerto Rico. Singapore data in 1982, showed a shift of dengue mortality from pediatric age to adult age group. In Bangladesh’s 2000 epidemic of DHF, showed 82% incidence amongst adult age group. This study shows that age incidence was highest between >18-40 years age groups, followed by >40-60. This shows similar trend that was started from 1980’s.

The incidence of dengue epidemics have been commonly associated with rainy season and the EI Nino phenomenon has been incriminated in the increases in certain vector borne diseases including dengue.^9^ A study from Puerto Rico from 1988 to 1992 showed weak relationship between mean temperature and incidence of dengue.^10^ The present study also shows that the time of epidemic from the middle of August to October (autumn season) and not occurring in rainy season. Thus, as concluded from the present study, factors related to crowd immunity, new serotype emergence or demographic transition influences the transmission.

The present study revealed that 79.52% of patients suffered from leucopenia (<5000/cc). However, Ratageri et al.^11^ showed only 21% of patients suffered from leucopenia and Banerjee et al.^12^ demonstrated no evidence of leucopenia in their patients. Subsequently, leucopenia in the current study may be due to virulent strain of dengue serotype.

The present study shows that during admission, 706 dengue patients (57.58%) expressed thrombocytopenia. But, within 2 days of infection, 350 more patients showed thrombocytopenia which increased the total number of patients of DHF to 1056 (86.13%). 82% and 96% of thrombocytopenic patients were reported in a study by Ratageri et al.^11^ and Banerjee et al.^12^ respectively. This thrombocytopenia may be attributable to decreased production of platelets due to bone marrow suppression^13^ and increased destruction, which may be immune mediated^14^ as a result of production of virus-antibody complexes and consequent complement activation. Again, release of high level of platelet activating factors by monocytes associated with secondary infection induce platelet consumption and increase adhesiveness of platelet with vascular endothelial cells produce thrombocytopenia.^15^

In DHF, there is evidence of plasma leakage as evidenced by detection of ascites and plural effusion by ultrasonography and x-ray chest. In the present study, 7.8% and 7.9% DHF patients suffered from ascites and plural effusion respectively, whereas Molta et al.^16^ detected 28%, 11.2% and 74.6% patients as right sided, bilateral plural effusion and ascites respectively by ultrasonography. Several observations suggest a massive T-cell activation during DHF, producing cytokines (interferon γ, interleukin 2, TNF α) and infected cell lysis by CD4+ and CD8+ dengue specific lymphocytes are responsible for plasma leakage. Cytokines may be released directly by macrophages/monocytes as a result of infection and indirectly due to interaction between infected cells and immune cells or both. A protein of 22-25 kDa responsible for capillary leakage has been evaluated in DHF patients.

## Conclusion

The seropositive DHF in the present study was 86.13% with predominant symptoms of headache, backache, nausea/vomiting, anorexia and loose motion. Females were anemic with low Hct value, but males showed raised Hct. This epidemic occurred in Autumn, male/female ratio was 1:1.08. About 7.8%-7.9% DHF patients showed evidence of plasma leakage. In this study, evidence of gross leucopenia (79.52%) may be due to virulent strain of dengue patients in this epidemic.
